# Supplementary figures and images for: HIV-1 diversity in viral reservoirs obtained from circulating T-cell subsets during early ART and beyond
Source: PLoS Pathog. 2024 Sep 18;20(9):e1012526. doi: 10.1371/journal.ppat.1012526 (PMC11410260; doi:10.1371/journal.ppat.1012526)

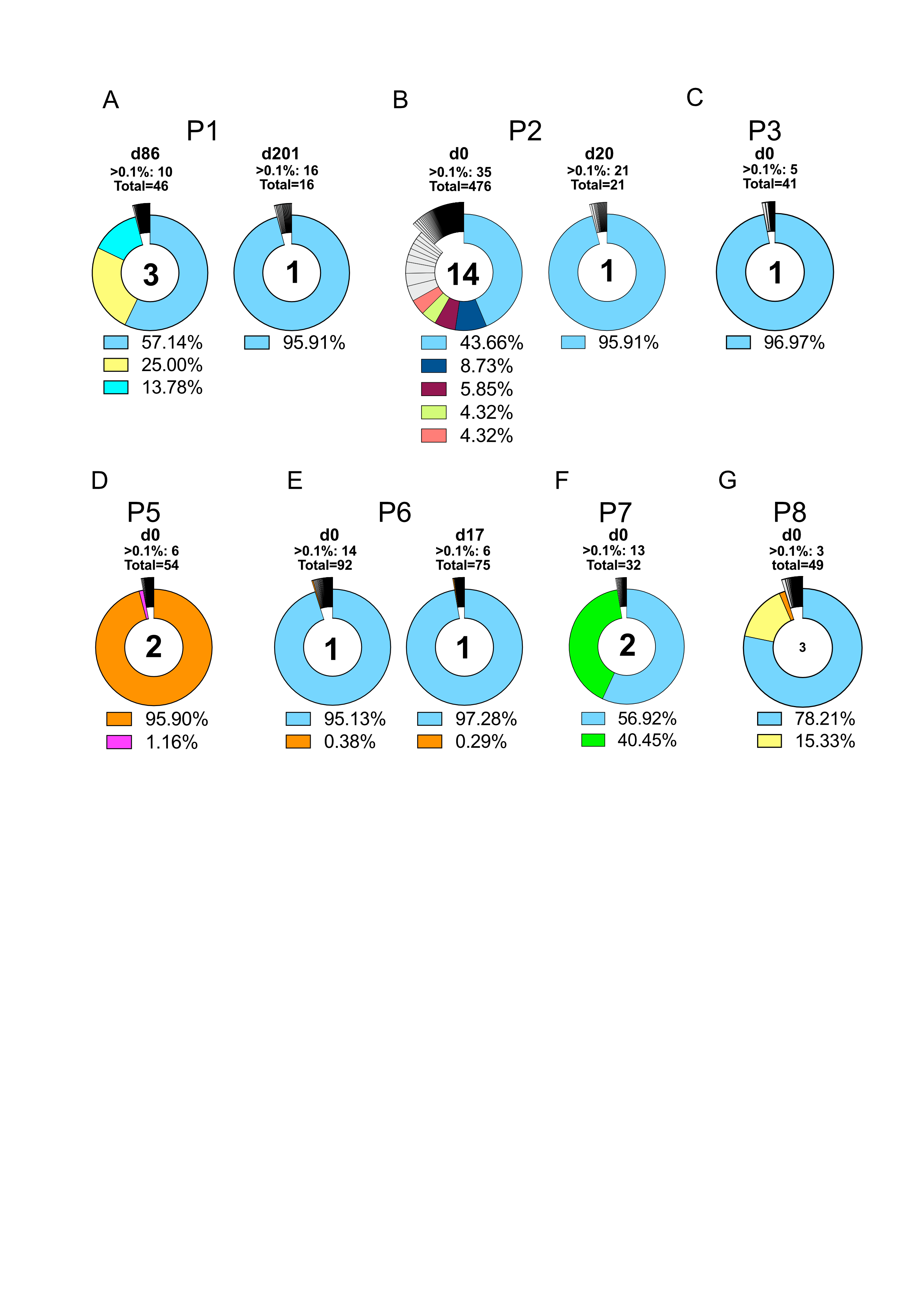

Supplement: S1 Fig — (A) P1. (B) P2. (C) P3. (D) P5. (E) P6. (F) P7. (G) P8. Pie charts depict HIV-1 RNA V3 loop diversity obtained by NGS. Sampling time points are shown above each pie chart (days during therapy). Variants with a frequency below 1% are combined in black, only the top 5 variants are depicted, each color representing one distinct virus variant. The total number of detected variants is summarized below each pie chart. (TIFF) [file ppat.1012526.s001.tiff]

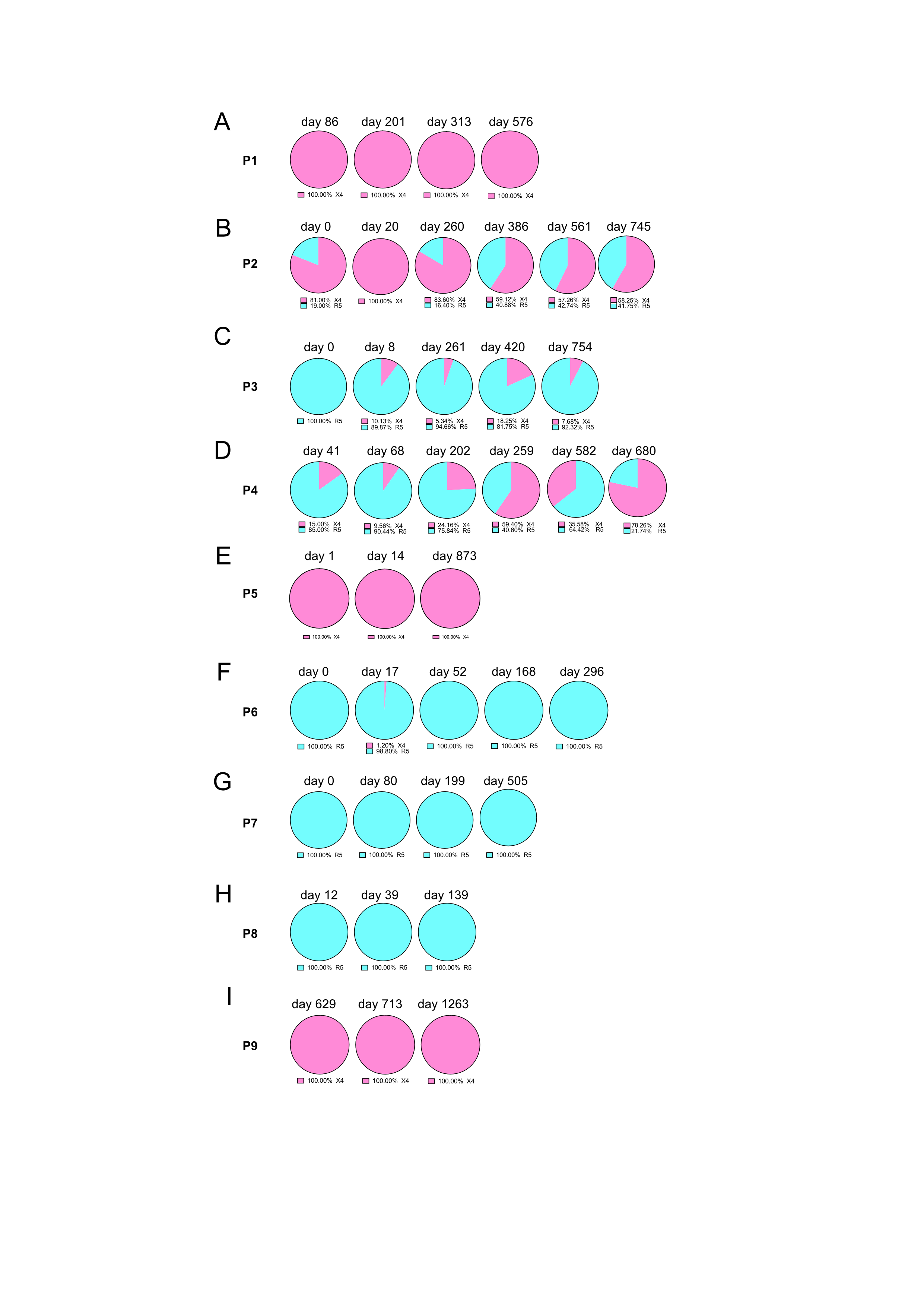

Supplement: S2 Fig — (A) P1. (B) P2. (C) P3. (D) P4. (E) P5. (F) P6. (G) P7. (H) P8. (I) P9. The frequency of R5- (cyan) and X4-tropism (pink) was determined by geno2pheno with an FPR cut-off of 10%. Only variants with a proportion ≥1% are shown. All individuals harbored B Subtype viruses. (TIFF) [file ppat.1012526.s002.tiff]

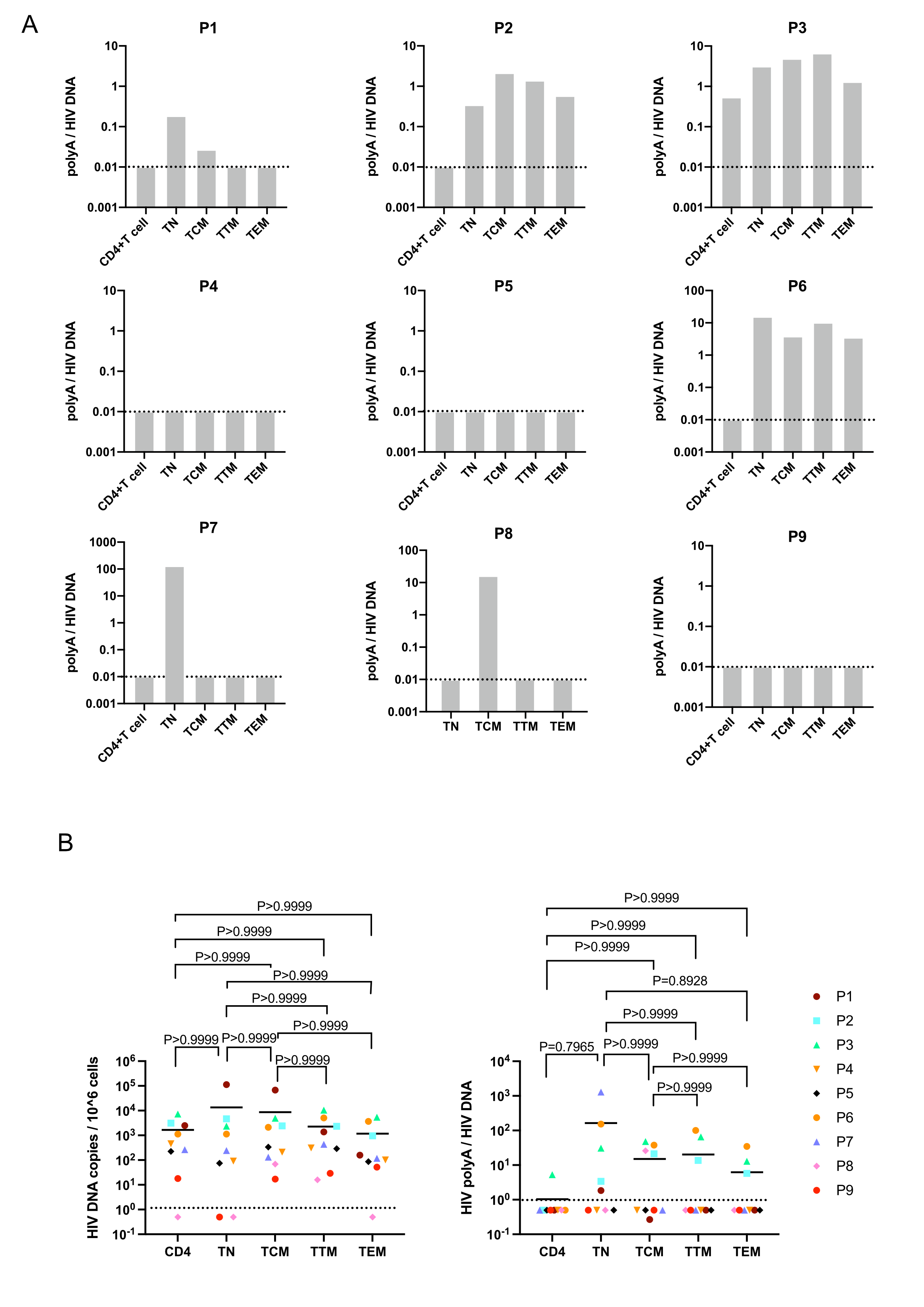

Supplement: S3 Fig — (A) Bar graphs of HIV poly-A loads / infected cells (HIV DNA) 5 days after stimulation. The black dotted line highlights the limit of detection. Corresponding individual IDs are above the respective bar plot. (B) Overall distribution of proviral DNA and cell-associated viral RNA per infected cell for each subset and all individuals. Black lines denote the mean value, and respective P values were determined by Two-way ANOVA and Bonferroni’s multiple comparison test. Individual IDs are shown in legend next to scatter plots. (TIFF) [file ppat.1012526.s003.tiff]

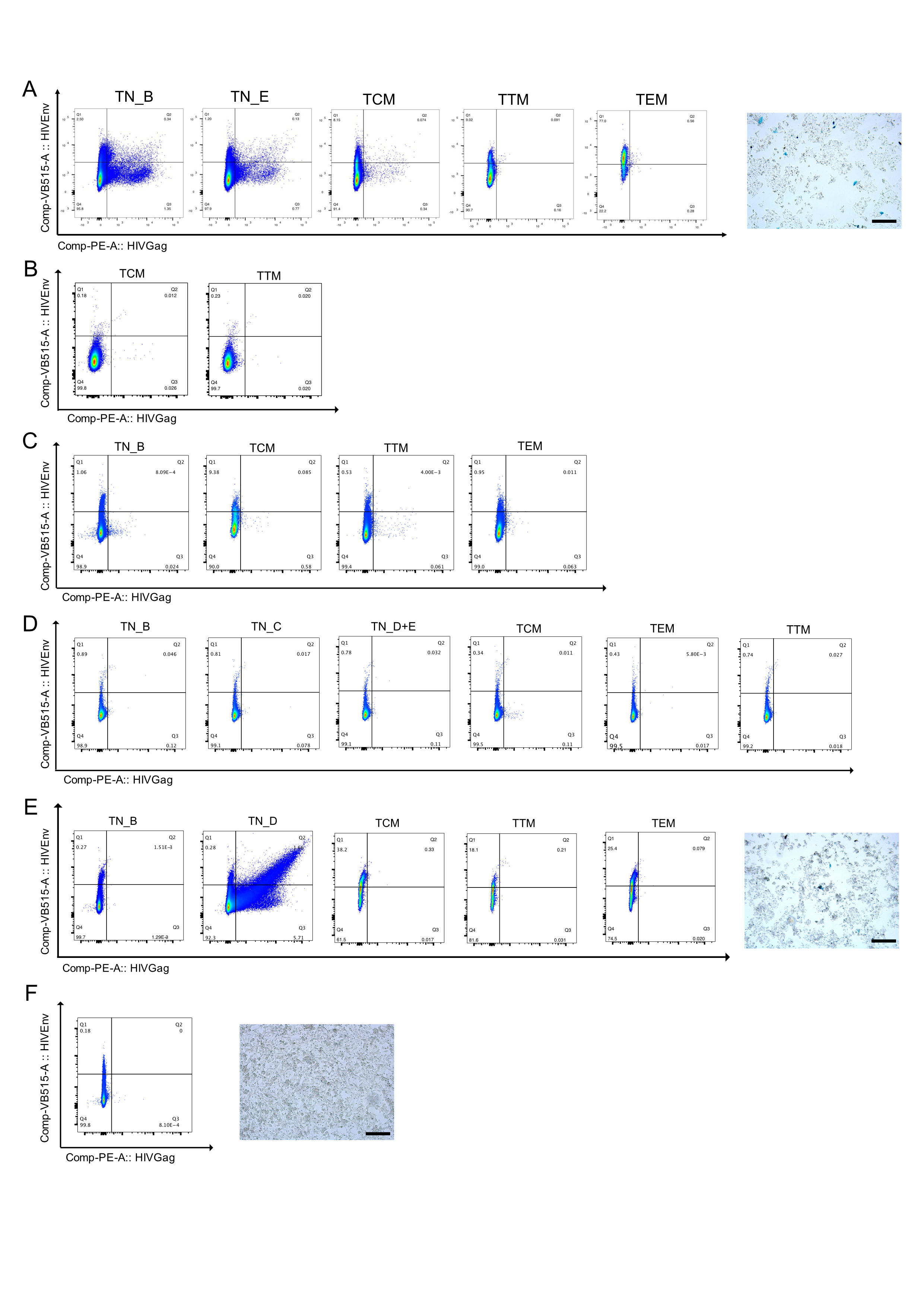

Supplement: S4 Fig — FACS plots show Gag vs. Env expression events in T cell subsets after in vitro T cell stimulation. Representative microscopic images show productive viral reactivation by LacZ (individual infection events in blue) after stimulation. (A) P1, (B) P3, (C) P4, (D) P6, (E) P7, (F) Uninfected PBMC as a negative control for GERDA and LacZ read-out of uninfected T cells post-stimulation (absence of blue cells). The scale bar is 250 μm. (TIFF) [file ppat.1012526.s004.tiff]

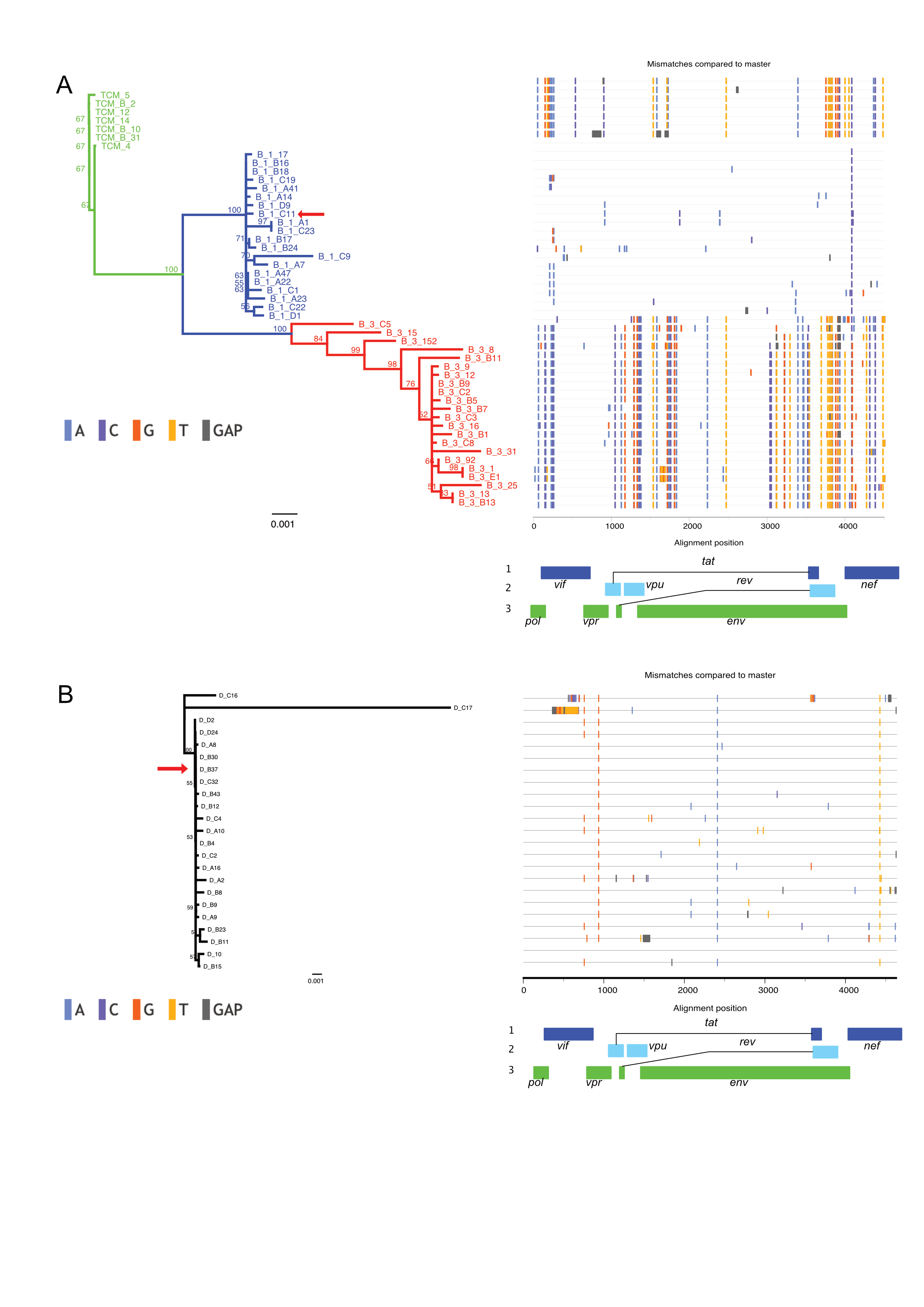

Supplement: S5 Fig — Phylogenetic trees and highlighter plots of positive outgrowth wells. (A) SGA viral variants identified for P1, in TN and TCM stimulated cultures (TN well NO.1: blue, TN well No.3: red, TCM well No.3: green); B_1_17 was selected as the master sequence). Amplification gaps at the 5’ end of B_1_C1, B_1_A7 or B_1_A14 were manually corrected, B_1_C11 was used to correct 5’ region (red arrow). (B) Individual SGA variants for P7, with D_10 selected as a master sequence, D_B8 and D_A2 were manually corrected, D_B37 was used to correct 5’ region (red arrow). (TIFF) [file ppat.1012526.s005.tiff]

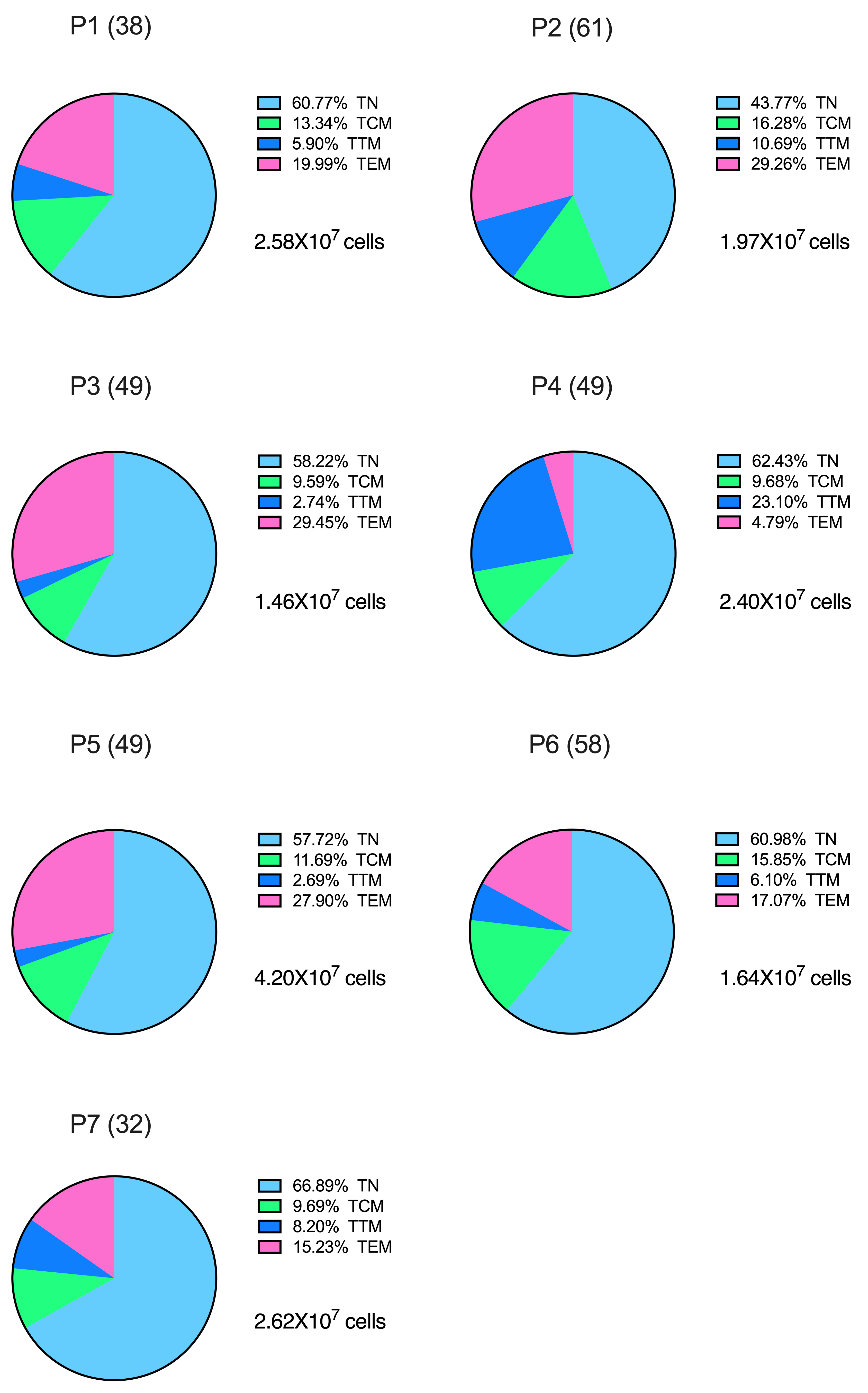

Supplement: S6 Fig — Distribution of all 4 sorted memory fractions. For each patient, ID and the respective age of the individual are given. % of each fraction is given in legend with respective memory assignment. The number next to the pie chart denotes the total amount of memory cells isolated for the respective patient. (TIFF) [file ppat.1012526.s006.tiff]
