# Supplementary material for: HIV-1 diversity in viral reservoirs obtained from circulating T-cell subsets during early ART and beyond
Source: PLoS Pathog. 2024 Sep 18;20(9):e1012526. doi: 10.1371/journal.ppat.1012526 (PMC11410260; doi:10.1371/journal.ppat.1012526)
Supplement: S1 Table — NA: not available; ND: not detectable; a re-initiation of ART; b first ART initiation (DOCX) [file ppat.1012526.s007.docx]

**S1 Table. Lab data of patient cohort.**

| **Group** | **Patient ID** | **Timepoint** | **Days since ART start** | **RNA VL (c/mL)** | **CD4 (cells/µL)** | **HIV-1 DNA/ 10^6^PBMC** | **HIV-1 PolyA/ 10^6^PBMC** | **HIV polyA/HIV DNA** | **TTV load** | **Recency/ History** |
| --- | --- | --- | --- | --- | --- | --- | --- | --- | --- | --- |
| High diversity | P1 | TP0 | -1 | 1,207,807 | 18 | NA | NA | NA | NA | unknown |
|  |  | TP1 | 4 | 34,500 | 107 | NA | NA | NA | NA |  |
|  |  | TP2 | 16 | 2,357 | NA | NA | NA | NA | NA |  |
|  |  | TP3 | 46 | 1,625 | 285 | NA | NA | NA | NA |  |
|  |  | TP4 | 62 | 519 | 318 | NA | NA | NA | NA |  |
|  |  | TP5 | 86 | 159 | 623 | 373 | <10 | NA | 3,085 |  |
|  |  | TP6 | 115 | 150 | 261 | NA | NA | NA | NA |  |
|  |  | TP7 | 201 | 55 | 316 | 240 | <10 | NA | 810 |  |
|  |  | TP8 | 313 | 36 | 348 | 984 | <10 | NA | 4,346 |  |
|  |  | TP9 | 424 | <10 | 408 | NA | NA | NA | NA |  |
|  |  | TP10 | 486 | <10 | 473 | 2,485 | <10 | NA | 11,743 |  |
|  |  | TP11 | 576 | <10 | 510 | 2,075 | <10 | NA | 2,543 |  |
|  | P2 | TP0 | -8 | 435,745 | NA | NA | NA | NA | NA | unknown |
|  |  | TP1 | 0 | 192,448 | 279 | 5,179 | 36,336 | 7.02 | 68,765 |  |
|  |  | TP2 | 20 | 379 | NA | 1,917 | 1,038 | 0.54 | 3,494 |  |
|  |  | TP3 | 48 | 33 | 506 | NA | NA | NA | NA |  |
|  |  | TP4 | 111 | 43 | NA | NA | NA | NA | NA |  |
|  |  | TP5 | 260 | <10 | 575 | 1,478 | 65 | 0.04 | 459 |  |
|  |  | TP6 | 386 | 230 | 536 | 3,141 | <10 | NA | 1,289 |  |
|  |  | TP7 | 447 | <10 | 687 | 923 | <10 | NA | 1,022 |  |
|  |  | TP8 | 561 | <10 | NA | 1,045 | 27 | 0.03 | 577 |  |
|  |  | T9 | 745 | <10 | NA | 660 | 1,466 | 2.22 | 3,461 |  |
|  | P3 | TP0 | 0 | 43,064 | 65 | 2,559 | 22,631 | 8.84 | 358,737 | Chronic  infection |
|  |  | TP1 | 8 | NA | NA | 65 | <10 | NA | NA |  |
|  |  | TP2 | 14 | 150 | NA | NA | NA | NA | NA |  |
|  |  | TP3 | 55 | <10 | 123 | 1,983 | <10 | NA | 39,584 |  |
|  |  | TP4 | 170 | <10 | 103 | 1,275 | 1,129 | 0.89 | 19,396 |  |
|  |  | TP5 | 261 | <10 | 110 | 1,270 | NA | NA | 1,556 |  |
|  |  | TP6 | 420 | <10 | 86 | 478 | <10 | NA | 11,114 |  |
|  |  | TP7 | 671 | <10 | 144 | 7,303 | 3,864 | 0.53 | 5,695 |  |
|  |  | TP8 | 811 | <10 | 133 | 1,008 | 340 | 0.34 | 538 |  |
|  | P4 | TP0 | -7 | <10 | 355 | NA | NA | NA | NA | unknown |
|  |  | TP1 | 0 | <10 | 672 | NA | NA | NA | 206 |  |
|  |  | TP2 | 41 | <10 | 813 | 36 | <10 | NA | 455 |  |
|  |  | TP3 | 68 | <10 | 714 | 506 | <10 | NA | 391 |  |
|  |  | TP4 | 202 | <10 | 758 | 135 | <10 | NA | 496 |  |
|  |  | TP5 | 259 | <10 | 646 | 231 | <10 | NA | 632 |  |
|  |  | TP6 | 315 | <10 | 542 | NA | NA | NA | NA |  |
|  |  | TP7 | 407 | <10 | 673 | 458 | <10 | NA | 718 |  |
|  |  | TP7 | 582 | <10 | 754 | 314 | 949 | 2.78 | 17,003 |  |
| Low diversity | P5 | TP0 | -21 | 7,670 | 433 | NA | NA | NA | NA | Chronic  infection |
|  |  | TP1 | -15 | 4,808 | 322 | 20 | <10 | NA | 142,673 |  |
|  |  | TP2 | 1 | NA | NA | 2,562 | NA | NA | 123,752 |  |
|  |  | TP3 | 14 | <10 | NA | 33 | <10 | NA | NA |  |
|  |  | TP4 | 127 | <10 | 630 | 13 | <10 | NA | 99,959 |  |
|  |  | TP5 | 204 | <10 | 553 | NA | NA | NA | NA |  |
|  |  | TP6 | 385 | <10 | 631 | NA | NA | NA | NA |  |
|  |  | TP7 | 546 | <10 | 754 | NA | NA | NA | NA |  |
|  |  | TP8 | 738 | <10 | 531 | 225 | <10 | NA | 36,297 |  |
|  |  | TP9 | 873 | <10 | 650 | 68 | <10 | NA | 40,022 |  |
|  | P6 | TP0 | -11 | 867,496 | NA | NA | NA | NA | NA | Acute  infection |
|  |  | TP1 | 0 | 143,033 | 511 | 4,414 | 85,033 | 19.26 | 147 |  |
|  |  | TP2 | 17 | 2,553 | NA | 3,743 | NA | NA | NA |  |
|  |  | TP3 | 52 | 142 | 807 | 1,297 | 418 | 0.32 | 328 |  |
|  |  | TP4 | 168 | 84 | 761 | 531 | <10 | NA | 84 |  |
|  |  | TP5 | 296 | NA | NA | 347 | <10 | NA | 221 |  |
|  |  | TP6 | 443 | <10 | 909 | 549 | 19 | 0.03 | 585 |  |
|  |  | TP7 | 808 | <10 | 999 | 1'131 | <10 | NA | 2,590 |  |
|  | P7 | TP0 | -39 | 5,972 | NA | NA | NA | NA | NA | Acute  Infection |
|  |  | TP1 | 0 | 25 | NA | 135 | <10 | NA | 37,787 |  |
|  |  | TP2 | 17 | <10 | NA | NA | NA | NA | NA |  |
|  |  | TP3 | 25 | <10 | 664 | NA | NA | NA | NA |  |
|  |  | TP4 | 80 | 47 | NA | 52 | <10 | NA | 3,845 |  |
|  |  | TP5 | 199 | <10 | 877 | 46 | <10 | NA | 9,210 |  |
|  |  | TP6 | 283 | <10 | 869 | 264 | <10 | NA | 1,595 |  |
|  |  | TP7 | 451 | <10 | 835 | 130 | <10 | NA | NA |  |
|  |  | TP8 | 505 | <10 | NA | 273 | <10 | NA | 26,942 |  |
|  | P8 | TP0 | -1 | 28,427 | 1,310 | NA | NA | NA | 1,390 | Acute  infection |
|  |  | TP1 | 12 | 90 | 1,928 | 276 | <10 | NA | NA |  |
|  |  | TP2 | 39 | <10 | 1,224 | 229 | <10 | NA | 71,276 |  |
|  |  | TP3 | 139 | <10 | 1,901 | 92 | NA | NA | 2,134 |  |
|  |  | TP4 | 241 | NA | NA | 43 | NA | NA | 760 |  |
|  | P9 | TP1 | 531 | <10 | 506 | ND | <10 | NA | 22,046 | Acute  infection |
|  |  | TP2 | 629 | NA | NA | 50 | NA | NA | NA |  |
|  |  | TP3 | 713 | <10 | 513 | 18 | NA | NA | 1,453 |  |
|  |  | TP4 | 811 | NA | NA | <10 | <10 | NA | NA |  |
|  |  | TP5 | 916 | <10 | 475 | <10 | <10 | NA | 4,990 |  |
|  |  | TP6 | 1,008 | <10 | 592 | <10 | <10 | NA | 4,759 |  |
|  |  | TP7 | 1,085 | <10 | 589 | 14 | <10 | NA | 1,975 |  |
|  |  | TP8 | 1,165 | <10 | 556 | 30 | <10 | NA | 1,198 |  |
|  |  | TP9 | 1,263 | <10 | 474 | 81 | <10 | NA | 3,611 |  |
|  |  | TP10 | 1,454 | <10 | 397 | 19 | <10 | NA | NA |  |
