# Supplementary material for: HIV-1 diversity in viral reservoirs obtained from circulating T-cell subsets during early ART and beyond
Source: PLoS Pathog. 2024 Sep 18;20(9):e1012526. doi: 10.1371/journal.ppat.1012526 (PMC11410260; doi:10.1371/journal.ppat.1012526)
Supplement: S2 Table — (DOCX) [file ppat.1012526.s008.docx]

**S2 Table. Clinical data of patient cohort.**

|  | **ID** | **Disease /**  **clinical condition** | **Months since diagnosis** | **Therapy** | **Months since Therapy** |
| --- | --- | --- | --- | --- | --- |
| High diversity | P1 | CMV | -4 | *bictegravir/emtricitabine/tenofovir alafenamide* | 0 |
|  |  | retinal HIV microangiopathy with cotton wool spots | 1 | *valganciclovir;*  *trimethoprim/sulfamethoxazole* | 5 |
|  |  | Pneumocystis jirovecii pneumonia | 1 | *valganciclovir* | 7 |
|  |  | Cervical lymph node infection with Mycobacterium avium | 2 |  |  |
|  | P2 | CMV | 1 | *bictegravir/emtricitabine/tenofovir alafenamide* | 0 |
|  |  |  | 2 | *dolutegravir/lamivudine* | 4 |
|  | P3 | Helicobacter pylori gastritis | -1 | *bictegravir/emtricitabine/tenofovir alafenamide* | 1 |
|  |  | HBV | 1 |  |  |
|  |  | Lues | 1 |  |  |
|  |  | subclinical Hyperthyroidism | 1 |  |  |
|  |  | Wasting-Syndrome | 1 |  |  |
|  |  | Penicillin allergy | 1 |  |  |
|  |  | CMV | 2 |  |  |
|  | P4 | HBV | -1 | *dolutegravir/lamivudine* | 0 |
|  |  | CMV | 1 | *doravirine/lamivudine/tenofovir* | 0.5 |
|  |  | Tuberculosis | 1 | *dolutegravir/ tenofovir alafenamide* | 15-16 |
|  |  |  |  | *dolutegravir/ tenofovir disoproxil* | 16 |
| Low diversity | P5 | CMV | 1 | *bictegravir/emtricitabine/tenofovir alafenamide* | 1 |
|  |  | HBV | 1 |  |  |
|  |  | Lues | 1 |  |  |
|  |  | asymptomatic Gonorrhoea | 10 |  |  |
|  | P6 | Chronic HCV  (treated with *elbasvir/grazoprevir*) | -36 | *bictegravir/emtricitabine/tenofovir alafenamide / darunavir / ritonavir* | 0 |
|  |  | Prostate carcinoma | 24 | *bictegravir/emtricitabine/tenofovir alafenamide* | 2 |
|  | P7 | Lues | -35 | Standard PrEP | 0 |
|  |  | Depression | -12 | *bictegravir/emtricitabine/tenofovir alafenamide* | 35 |
|  |  | pathological distal tibial shaft fracture | -4 |  |  |
|  |  | HAV | -1 |  |  |
|  |  | CMV | 1 |  |  |
|  |  | COVID19 | 2 |  |  |
|  |  | ESBL Shigella sonnei | 10 |  |  |
|  |  | Chlamydia | 23 |  |  |
|  | P8 | Lues | -20 | *bictegravir/emtricitabine/tenofovir alafenamide* | 1 |
|  |  | recurrent Herpes labialis | NA |  |  |
|  | P9 | Toxoplasmosis | 1 | *abacavir/dolutegravir/lamivudine* | 0 |
|  |  | CMV | 1 | *dolutegravir/lamivudine* | 45 |
|  |  | Tuberculosis | 1 |  |  |
