# Supplementary material for: HIV-1 diversity in viral reservoirs obtained from circulating T-cell subsets during early ART and beyond
Source: PLoS Pathog. 2024 Sep 18;20(9):e1012526. doi: 10.1371/journal.ppat.1012526 (PMC11410260; doi:10.1371/journal.ppat.1012526)
Supplement: S3 Table — Representative NGS data of most detected proviral V3 variants of all individuals at the first sampling time point. Amino acid changes of individually aligned V3 variants are shown in black and underlined in bold. (DOCX) [file ppat.1012526.s009.docx]

**S3 Table. V3 loop NGS data.** Representative NGS data of most detected proviral V3 variants of all individuals at the first sampling time point. Amino acid changes of individually aligned V3 variants are shown in black and underlined in bold.

| Group | ID | Day after diagnosis | V3 Loop |
| --- | --- | --- | --- |
| high diversity | P1 | d86 | CTRPTNNTRTRTAIGQGQVWYRREGIIGDIRKAYC |
|  |  |  | CTRPTNNTRTRTAIG**P**GQVWYRREGIIGDIRKAYC |
|  |  |  | CTRP**A**N**K**TR**I**RTAIGPGQVWYR**QGD**IIGDIRKAYC |
|  |  |  | CTRP**G**NNTRTST**S**IGPGQVWYR**QGD**IIGDIRKAHC |
|  | P2 | d0 | CIRPGNNTRRSVRIGPGQVFYATGEITGDIRKAHC |
|  |  |  | CIRPGNNTRRSVRIGPGQVFYATGEI**I**G**N**IRKAYC |
|  |  |  | CIRPGNNTRRSVRIGPGQVFYATGEI**I**GDIRKAYC |
|  |  |  | CIRPGNNTRRSVRIGPGQVFYATGEI**I**G**N**IR**Q**AYC |
|  |  |  | CIRPGNNTRRSVRIGPGQVFYATG**K**ITGDIRKAHC |
|  | P3 | d0 | CTRPGNNTRRSIHIGPGKAFYTSEITGDIRQAHC |
|  | P4 | d41 | CTRPSNNTRQGVHMGPGQVFYKTGEIIGDIRKAYC |
|  |  |  | CTRPSNNTRQGVH**I**GPGQVFYKTGEIIGDIRKAYC |
|  |  |  | CTRPSNNTRQ**S**VHMGPG**S**VFYKTGDIIGDIRKAYC |
|  |  |  | CTRPSNNTRQGVH**I**GPGQVFY**R**TGEIIGDIRKAYC |
| Low diversity | P5 | d1 | CTRPSNNTRSSIRIGPGQVFYKTEDIIGDIRKAFC |
|  |  |  | CTRPSNNTRSSIRIGPGQVFYKT**G**DIIGDIRKAFC |
|  |  |  | CTRPSNNTRSSIRIGPGQVFYKT**GAI**IGDIRKALC |
|  |  |  | CTRP**F**NNTRSSIRIGPGQVFYKT**G**DIIGDIRKAFC |
|  | P6 | d0 | CTRPNNNTRKSIHMGFGKTFYATGEIIGDIRQAHC |
|  | P7 | d0 | CTRPNNNTRESISIGPGRAFFATGDVIGDIRQAHC |
|  | P8 | d12 | CIRPNNNTRKSIHIGPGQAFYATGDIIGDIRQAHC |
|  |  |  | **G**IRPNNNTRKSIHIGPGQAFYATGDIIGDIRQAHC |
|  | P9 | d629 | CTRPFNNTRSSIRIGPGQVFYKTGAIIGDIKKAYC |
