# Supplementary material for: HIV-1 diversity in viral reservoirs obtained from circulating T-cell subsets during early ART and beyond
Source: PLoS Pathog. 2024 Sep 18;20(9):e1012526. doi: 10.1371/journal.ppat.1012526 (PMC11410260; doi:10.1371/journal.ppat.1012526)
Supplement: Attachment — Submitted filename: renamed_15b1d.pdf [file ppat.1012526.s010.pdf]

HIV-1 diversity in viral reservoirs obtained from circulating T-cell subsets during early ART and beyond

Zhang and Otte…Klimkait, and the Swiss HIV Cohort Study

Summary of manuscript:

The manuscript here by Zhang and Otte *et al*. examined Env V3 diversity in various T cell subsets during early ART to characterize the dynamics over time. Very interestingly, the authors found a strong association between the Torque-teno-virus (TTV) and levels of HIV-1 plasma viremia and viral transcription as measured by polyA. Additionally, the level of Env V3 diversity appeared to reflect TTV load and was dynamic pre-ART and following therapy. The authors bring attention to a possibility of utilizing TTV load for immune status as it relates to HIV infection.

While intriguing, there were several major concerns that should be addressed in order to strengthen the manuscript as there is a great amount of potential (detailed below). Further, please be mindful of Person First language by replacing instances of "patient".

Specific comments (major and minor)

Abstract:

Generally, the abstract reads well. The sentence in Line 25, "Complementing information was obtained…" seems worded oddly, possibly consider re-wording. While the abstract does not serve to explain all findings, the connection to TTV is lacking and/or weak. I found it slightly distracting as I had never heard of this virus before and thus, any clinical relevance was lost.

Introduction:

- Line 55: "HIV-1 virus reservoir", the word 'virus' is repetitive to 'HIV'.
- Line 57: Suggest changing to just "ART" as it is all highly active and capable of preventing ongoing rounds of viral replication.
- Lines 59-63: Suggest rewording and making more succinct. It is understood what the authors are trying to say but the execution is disjointed. In addition, while of course rare, proviral eradiation has been shown to be possible through the Berlin Patient and other hom. CCR5delta32 alloHSCT.
- Line 65: "Infection with HIV-1" suggest changing to "HIV-1 infection".
- Line 65-68: Words like 'significant' and 'short time' feel non-descriptors. Consider Coffin (1995) PMID 7824947 as it also describes how the population size is a key factor.
- Line 78: Avoid "patient" and use People First language.
- Lines 80-83: While still the introduction, include how you are measuring diversity.
- Line 88-89: The TTV load correlate to HIV transcription as a suitable marker for clinical manifestation is too strong (discussed further in later comments), suggest modifying.

Results:

- Lines 99-100: I would specify “different phases of HIV infection”. Acute vs chronic, Fiebig staging, etc would be useful. Take the time to mention STable 1 since it would help the reader greatly.
- Line 111: “chronic control situation” is worded oddly.
- Line 139-143: In relation to STable 2, please double check spelling, proper italicization when appropriate, and for drug names for ART and other treatments use the international naming (e.g. valganciclovir instead of valcyte).
- Line 150-154: Consider adding that viremia was suppressed to undetectable levels in donors following ART initiation, or something to that effect, it is worded oddly. It is mentioned that viral transcription was detected even post-ART for “long periods”, is there a reason that the authors might have thought otherwise since stochastic reactivation of infected cells contributes to residual viremia. The last sentence about transcriptional activity would be better connected without the statement of suppression of viremia between them.
- Lines 159-: It is a little misleading to state you are interested in understanding viral dynamics in the long-term reservoir on ART. Perhaps something was missed here. But once on ART, ongoing rounds of viral replication is halted, so there would be a lack of evolution, and studies by Sarah Joseph and Zabrina Brumme have independently shown that the reservoir post-ART is primary reflective of the composition right before ART initiation—which the author’s data does support in Line 177-178. The idea of “long-term” would be >4 years, perhaps even longer (>7 years), or even longer. Donor 9 is the only that is even close to 4 years. It is also known that diversity is less in those who started ART during acute infection, in part due to the presence of a smaller reservoir size. It is not clear if these factors were considered. It was not clear in SFig 1 what “total” referred to, assuming total sequences obtained?
- Line182-188: What are the subtypes of these donors? Assuming subtype B. But including that would be helpful to place the tropism prediction into context since Geno2Pheno doesn’t work well for all subtypes. Have the authors considered also using PhenoSeq (https://tools.burnet.edu.au/phenoseq/) to support their predictions through the use of two software tools? The authors mention that in P4 there was a “decrease” in X4 tropic viral variants, have these numbers been normalized to how many sequences were obtained (i.e. time point 1 has 50 sequences but time point has 500, the sampling depth at TP2 is 10x greater).
- Line195-203: Suggestion to clarify Figure 2 legend. In reading the Results text it was clear that the V3 sequences were from the proviral population but the legend begins talking about post-stimulation which is confusing. Also, are the Figures labeled correctly since there is “Fig 1B”? Also suggest adding the number of sequences obtained as a total for each subset and normalized if possible. If the bulk CD4 was used as a comparator, it would be nice to see some sort of p-value to understand how large some of these clones are between subsets compared to the bulk CD4.
- Line 222-226: In following the author’s Methods section related to the measurement of polyA HIV transcripts there are two points that would be important to bring up. The

reference (#30) for the method of HIV polyA RNA is the reference for TTV detection. I have assumed the authors are using an HIV-1 gene-specific polyA primer for the purposes of the results. While the amount of RNA is normalized to CCR5, it would be important to normalize to the number of infected cells. Without knowing how many infected cells were assayed, in the cases where it wasn't detected, enough infected cells might not have been assayed. Also it might be worth noting that depending on the length the RNA sat in the -80C, you can have degradation starting after several weeks/months.

- Line 230: For the IUPM 95% Cis only need to report to the 2nd decimal place (also Line 243). Out of curiosity, and might be worth including, was the viral outgrowth performed at limiting dilution, or multiple bulk wells plated? Could be worth including as well to be clear whether the outgrowth assay was a quantitative VOA—especially since you state QVOA in Line 249. In Line 245, I think the authors mean S2B not S2F.
    - After reading through the legends and methods, a suggestion that would benefit your phylogenetic trees would be the following. The gaps are artificially leading to long branches. If possible, either gap-strip manually, then reconstruct the trees. Or if using MEGA, try performing a 'complete deletion' NJ. Also change the scale to nucleotide so the reader can easily determine how many bases different the sequences (or populations) are from one another. Also looking at S5B, the viruses that have the common "A" are probably clonal. The random 1-2 nucleotides observed to give them little branches is probably fuzz from introduced sequencing error.
- Line 256: "derailment" is an odd word to use here.
- Line 258: The authors need to define what is being set as "high variability" as it is quite ambiguous. If the cut-off is >5 viral variants, please state that, or if the authors can develop a statistical determination, use that as well. But as a reader, knowing what exactly is meant is crucial.
- Line 270: For reported p-values in-text and in the figures, too many sig figs are used, the authors can simplify them.
- Line 263-280: The inverse correlate of TTV and immune status is fascinating. As stated previously, the leap to an indicator and having clinical significance is a little strong and does require more work. The biggest concern I have here is that if you sampled TTV in a person living without HIV overtime, would the dynamics of TTV load be similar as those in persons living with HIV—since it is highly prevalent? And if that work has already been done, the authors should highlight that before going into potential differences of high and low diversity groups. It would also benefit to demonstrate data where TTV is not detected. It begins to fall outside the scope of this article, but it feels like a strong emphasis is drawn to it. By including additional experiments such as suggested, understanding the possible correlation and overall biological significance can better be explored.

Discussion:

- Line 309-315: As stated, prior, the strong language of TTV is too much. The authors connect alloHSCT with TTV but the reference to Einkauf *et al*. does not seem appropriate here.
- Line 348-354: This comment originates from the introduction, and this appears to be a “good location” to discuss. The authors are correct in their assessment on central memory. Have the authors normalized the different subsets since central memory is much larger than effector memory? In the introduction, it was expressed as though this was a “new” finding but Chomont…Sékaly (2009) and Kwon…Siliciano (2019) also highlighted these points regarding central memory. This would go further to help Lines 254-259 as well.
- Line 385-387: This sentence does not make, the concept of what the authors are trying to say is there but could use some work.

Methods:

- If the authors can include the purity% of their subsets as a supplemental table or elsewhere, it would be a good addition. Or a general statement of the >x% purity etc.
- Could more be added about the pipeline for NGS? And the Illumina runs? Assuming dogtags are used, is it possible to bin sequences and generate majority-rule consensuses similar to what has been reported previously the Swanstrom group? Overall, since Geneious’s assembly methods can vary, some additional detail would be helpful.
- For the statistics, is there a particular reason the authors did not use Spearman—seemed more logical. Also, it would be good to state what type of “regression” was performed. Was the data normal so that a linear regression (assuming that was the method) made sense without data transformation? For simplicity, you could easily remove the 95%CIs of the regression.
