# Supplementary material for: HIV-1 diversity in viral reservoirs obtained from circulating T-cell subsets during early ART and beyond
Source: PLoS Pathog. 2024 Sep 18;20(9):e1012526. doi: 10.1371/journal.ppat.1012526 (PMC11410260; doi:10.1371/journal.ppat.1012526)
Supplement: Attachment — Submitted filename: 20240709_Point-by-point response.docx [file ppat.1012526.s011.docx]

Dear Editor,

We thank the Reviewers for the critical and constructive evaluation of our work. We found many suggestions very helpful for improving the manuscript and have tried to resolve all unclear or unaddressed points by re-analyzing the data or by amending crucial information that had been missing before.
We kept the format of the “Reviewer’s Response to Questions” that was sent to us by Mail.

Please find below in the point-by-point review the reviewers’ queries in black and the text of our responses in blue.

Responses to Questions

**Part I - Summary**
Please use this section to discuss strengths/weaknesses of study, novelty/significance, general execution and scholarship.

**Reviewer #1**: The manuscript here by Zhang and Otte et al. examined Env V3 diversity in various T cell subsets during early ART to characterize the dynamics over time. Very interestingly, the authors found a strong association between the Torque-teno-virus (TTV) and levels of HIV-1 plasma viremia and viral transcription as measured by polyA. Additionally, the level of Env V3 diversity appeared to reflect TTV load and was dynamic pre-ART and following therapy. The authors bring attention to a possibility of utilizing TTV load for immune status as it relates to HIV infection.

While intriguing, there were several major concerns that should be addressed in order to strengthen the manuscript as there is a great amount of potential (detailed below). Further, please be mindful of Person First language by replacing instances of “patient”.

Thank you for the important reminder of respectful language use: Any use of the term “patient” has been changed to “individual” or “study participant” throughout the text. (the phrase is now only found in the title of six publications in the list of references)

**Reviewer #2**: Zhang et al characterize HIV proviral (env) diversity between ART initiation and suppression in 8 participants (3 early infection, 2 chronic infection, 3 unknown), plus a ninth participant who was on long-term ART at study entry. The authors combine various techniques, including total proviral load measurements (single-target), within-host proviral diversity estimates obtained through bulk (NGS) sequencing of the V3 loop region, cell-associated poly-A RNA loads, along with single-genome sequencing (3' half) and viral outgrowth assays on sorted CD4+ T-cell subsets, to characterize the replication-competent HIV reservoir and overall proviral pool during this key phase.

This is an interesting question and valuable analysis, but the manuscript is unclear and confusing in parts, and contains what appear to be errors and mis-statements, which made it a challenge to read and understand. Also, the major stated conclusions of the study are not entirely novel (e.g. early ART limits HIV diversity, see PMID 24277811, 28046096) or potentially flawed due to analysis issues. Namely, the conclusion that replication-competent viruses were exclusively observed in TN or TCM is based on analysis of insufficient biological material, while the conclusions that poor immune status correlates with HIV diversity, and that TTV loads correlate with HIV transcription on ART, were interpreted from data that were incorrectly analyzed. While the conclusions may indeed be correct, as they are consistent with the reports of others, these issues represent major limitations.

All points of criticism raised here, especially on potentially inappropriate analyses, will be discussed in detail in our responses below.

In addition, we have re-worked the phrasing to increase clarity and readability of the text.

**Reviewer #3**: This study aims at describing the HIV-1 reservoir diversity (based on diversity of the V3 loop) and infection competence in 9 individuals on ART. The main finding is that greater diversity is observed in TTMs and TEMs and that infection competent viruses are identified only in TCMs and TNs with low diversity. The study also shows inverse correlation between proviral diversity, and immune competence and a correlation between HIV and TTV viral loads.
The methods and analyses used are appropriate for addressing the research questions. A possible limitation would be the sole focus on the V3 region as representative of env diversity. The findings are an important addition to our understanding of HIV intra-host dynamics and evolution as they suggest a rudimentary difference between HIV diversity in different T cells.

This reviewer raised the potential “limitation” of restricting the analysis to the V3 region of envelope. However, we deliberately chose this region, well established in the field as highly variable region. Due to its high variability, we should be able to follow the viral dynamics and even minor sequence changes.

Suggested minor revisions:
line 175: “Although in P5, plasma virus was fully suppressed by day 477 of therapy, intracellular HIV transcripts remained detectable at days 561 and 745 (Figs 1A and 1B).”

According to figure 1B there is no intracellular mRNA detected in P5.

We thank the reviewer for pointing out the typo: The sample was corrected to “P2”; to enhance the text low, this paragraph was moved to line 180.

Line 200: “For P1, we detected small proviral minorities in bulk CD4, TN, or TCM, e.g. on days 86 and 201 during therapy(Fig 1B) that were predominant with 90.4% of the virus population in TTM (Fig 2A). The same phenomenon was seen in the TTMs of P2, P3, P4, and P7, and in the TEMs obtained from P1, P2, P4, P5 and P6, (Fig 2A).”

This statement is somewhat inconsistent with the results. The first sentence is a little confusing, while it is true that there are small minorities in bulk CD4, TN and TCM, the time that these minorities were identified by NGS was according to the figure 2 legend, 5 days post-stimulation via QVOA which according to figure 1 was performed for P1 after day 400, therefore it is confusing that the authors continue with "e.g., on days 86 and 201" which were measured by NGS (presumably from bulk CD4+ T cells). Then the authors write that these minorities were predominant with 90.4% of the virus population in TTM (of P1) which is consistent with the figure, however in the following sentence they write that "The same phenomenon was seen in the TTMs of P2, P3, P4, and P7, and in the TEMs obtained from P1, P2, P4, P5 and P6", but this statement is inaccurate, because in these other participants the minority sequences do not reach 90% in TTMs in P2 and P3, and also they do not reach 90% in the TEMs of P1, P2, P5 and P6. To support a claim about increased diversity in TTMs and TEMs it would be beneficial to add a bar plot comparing the percentage of minority sequences in the different T cells, in each participant.

Thanks for pointing out this misleading sentence. We have now removed the term “increased diversity”, which would imply a consistently growing number of viral quasispecies. To fit the context better, we have exchanged the term for “shifted diversity”, to rather indicate that the sequence composition of the viral quasispecies has CHANGED (without indicating an increase in number).

Rephrased (changes in *italics) text line 205*:

“For P1, we detected *in TTMs a predominant proviral variant (90.4% of the whole viral population) that was only found as a* small proviral minority in bulk CD4, TN, or TCM *(Fig 2A). This small minority was already present for bulk proviral NGS sequencing during earlier treatment periods,* e.g. on days 86 and 201 *after* therapy (Fig 1B). *The same phenomenon of proportionately higher predominating variants was seen in the TTMs of P2, P3, P4, and P7, and in the TEMs obtained from P1, P2, P4, and P5 (Fig 2A).*”

Line 209 (Figure 2 legend): “…B,C: NGS V3 loop diversity of viral RNA from QVOA positive wells. (B) Reactivated virus of TN and TCM form P1, C: Reactivated virus of TN form P7.”
For consistency, C should be in parenthesis.

The legend of Figure 2 has been corrected accordingly.

line 224: “In the T-cell populations from 4/9 individuals (P4, P5, P9) no viral transcription could be induced (S3A Fig) and overall, levels of proviralHIV-1 DNA as well as viral transcripts were similar in every T cell subset (S3B Fig).”
Should be changed to 3/9 individuals.

Section has been corrected as pointed out by the reviewer.

In Fig S5A positions ~600 and ~1000 seem to be shared between the red and blue clades. This might be an indication of a recombination event in this region.

We thank the reviewer for pointing this out. A statement has now been added to the text line 247:

“In Fig S5A, the first 1000 bp of the red and blue clades are almost identical, suggestive of a potential viral recombination event.”

General comment:
Although the paper cites a couple of papers that also used V3 as a marker for diversity, it is not clear how representative the V3 diversity of other env regions? Is it possible that increased V3 diversity is the result of V3 targeting antibodies, and that in people with low V3 diversity other regions of env are targeted by antibodies and are therefore more diverse? It might be worth mentioning in the discussion.

Thanks for this valuable additional point, which prompted us to add a short paragraph to the discussion, text line 384:

“Furthermore, our sequencing analysis mainly focused on the variable V3 Env region as it has been reported to be under continuous rearrangement, which can lead to events such as genomic mutations that can escape V3-targeting antibodies. We therefore interpret the observed enhanced dynamics rather as an advantage for emerging viruses. The NGS data are further supported by SGA sequencing data that analyses the entire 3’ half of the HIV-1 genome. At the same time, a continuing intra-host evolution under (fully suppressive) therapy is not expected in the peripheral blood.”

**Part II – Major Issues: Key Experiments Required for Acceptance**
Please use this section to detail the key new experiments or modifications of existing experiments that should be absolutely required to validate study conclusions.
Generally, there should be no more than 3 such required experiments or major modifications for a "Major Revision" recommendation. If more than 3 experiments are necessary to validate the study conclusions, then you are encouraged to recommend "Reject".

Reviewer #1: Please see full review.

Reviewer #2: I expand on the last two comments below:

1. It appears that the CD4+ T-cell subsets sorted for the QVOAs were obtained from 40ml of blood only. This is insufficient. It is therefore not surprising that viral outgrowth was only successful in 2 of 9 participants, where only Tn and Tcm yielded viral outgrowth. This is likely because these are typically the largest subsets, so possibly the only ones in which sampling was adequate to identify something. What were the bulk QVOA measurements for the study participants? The QVOA values obtained from Tn and Tcm subsets of the two successful participants were all < 1 IUPM, which further supports the need for much larger amount of biological sample to have been assayed.

We fully agree with the reviewer that a larger cell sample could have significantly increased cell numbers needed for a most reliable analysis of the respective subpopulations. However, due to an unfortunate limitation for the sample volume of the current SHCS ethical approval, we were not permitted to obtain and utilize more than 40 mL per time point and individual.

Based on this inherent limitation, we conducted all possible sorting steps with an “untouched” protocol. This was to ensure that the original cell populations would be sorted prior to any cellular activation or skew in the cell populations.

Morover, considering the proviral and transcriptional burden of each subset after normalization to cell counts or infected cells (highlighted in Figure S3B) there was no significant enrichment in any of the sorted fragments. We have added a supporting Figure highlighting the relative contribution of each subset to the memory T cell compartment. TN (mean 58.68%) is more abundant, but TCM (mean 12.30%), TTM (mean 8.49%) and TEM (mean 20.53%) are equally represented. Thus, TTM or TEM have the same “chance” for a successful viral outgrowth as TCM.

We principally agree that a larger number of cells from a larger volume of blood would increase the chance for a positive VOA; we have amended the current data as a supplemental Figure Fig S6 to the manuscript:

For additional information, we have now included a clarifying statement in the discussion section, text line 379:

“Besides using a larger number of samples, the analysis would principally benefit from using higher blood volumes to increase the chances of reactivating dormant proviruses. Yet, the Swiss cohort ethical approval only allows full blood volumes of up to 40 mL. Irrespectively, TCM (mean 12.30%), TTM (mean 8.49%) and TEM (mean 20.53%) are similarly represented with equal normalized proviral and transcriptional loads (Figs S3B and Fig S6).”

2. The data in Figure 3 are analyzed incorrectly, as participants are represented multiple times on each plot. It looks as if as if the authors plotted all participants' longitudinal measurements as independent data points, and applied statistics, which is in violation of the i.i.d principle. There are dozens of data points in Figures 3A-C, for example, even though there are only 9 participants. Any analysis exploring correlations between participant-derived features (e.g. immune status and reservoir diversity) should represent each participant only once (or if it is a longitudinal analysis, with each participant measured once at each time point and the points connected). This could be done by, e.g. averaging the CD4 counts and diversity measurements for each participant. The same limitations apply to the analysis of TTV loads. This calls into question the validity of the conclusions made from all data in this figure.

We would like to thank the reviewer for highlighting this very relevant aspect. All data of Fig. 3 have now been re-analyzed using averaged values. It turns out that neither lymphocyte counts nor CD8 counts show any difference among the two viral diversity groups. CD4 counts do not reach significance but a clear trend is visible for all three time intervals. TTV levels appear to drop faster in the high diversity group but do not reach significance likely due to missing measurements. Missing values for the poly-A quantification do further not allow any major conclusions on the differences among the two groups. A bigger cohort might eventually reveal statistical significance as raised in the discussion section lines 379 ff.

Other comments:

1. How was it determined that, of the 9 participants, 3 were diagnosed in primary and 2 in chronic infection (lines 105-107)? This needs to be described. The sentence prior (line 102-104) suggests that infection recency was predicted based on the participant proviral load measurement - but if this were the case, this would represent a major limitation (in addition, there may be an error in this sentence, which states that "a high proviral DNA load would be indicative of an early infection phase", which seems at odds with the evidence that early ART limits reservoir size).

All recency data were reported as per medical records of the clinical center. This information has now been added to the results section and to the M&M section “Study participants” line 416:

“All infection recency information was obtained through the clinical records available at the SHCS centers.”

We agree that the recency comment on a “high proviral load” may be counter-intuitive and could be misleading to the reader. We therefore removed this uncritical sentence from the manuscript.

2. Lines 145-147 state that proviral DNA loads and poly-A RNA levels were determined "at the time of diagnosis and at each follow-up visit" but this appears to be incorrect, as sampling from P1, P4 and P8 appear to be exclusively post-ART, and others appear to start on day of ART start. Please clarify.

Thanks for the point raised here. The sentence was rephrased and adjusted to accommodate the inclusion of P1, P4, and P8. Changes in *italics, text line 150.*

“To assess viral dynamics and intracellular activity, proviral DNA loads and cell-associated poly-adenylated (pA) RNA loads were determined at *the earliest time point available at or shortly after* diagnosis and at each follow-up visit (Fig 1B)”

3. It appears that, for the analysis of proviral DNA diversity in T-cell subsets presented in Figure 2, that the cells were cultured prior to analysis. Why was this done, and could this affect the distribution of variants observed?

Due to the limited amount of available cell material, cells were first amplified in a defined fashion by CD2, CD3 and CD28 stimulation before analysis. We are fully aware that this might introduce a certain skew, but use of a defined procedure for every sample should allow a reasonable side-by-side comparison (rather than a true reflection of the in vivo situation). By using the established and accepted procedure of CD2, CD3 and C28 stimulation, ≥95% of the cells were (re-)activated.

Furthermore, with this procedure we did not expect any principal distortion of the viral reservoir, since no external cell-free virus from other compartments was present in the samples after cell separation, which could have been expanded by the procedure.

4. Related to this, it appears that the data in Figure 2 were derived from a single time point, but this is not stated, and it is also unclear whether this is a different time point than those shown in Figure 1. Also, lines 198-199 state that "the viral reservoir remained largely stable in Tn and Tcm compartments compared to the bulk PBMC" but this is misleading as it suggests stability over time. But, the subsets analysis was cross-sectional.

The information about the time points used has now been added to the figure. For clarity, we rephrased the sentence on TN/TCM and bulk variant composition. Changes in italics, text line 203:

“*Proviral* NGS *analysis* showed that in most individuals the *repertoire of identified variants in* the TN and TCM compartments *was largely similar to that identified for the bulk CD4+ T cells or for earlier time points* (Figs 1B and 2A).”

5. Figure 2 interpretation: text line 203. The inclusion of P6 in this group is not supported by the data, as the variant is not "predominant" in Tem by any means.

The reviewer rightly pointed this mistake out! Individual P6 has now been removed in this section.

6. Figure S3 appears to be erroneous. S3B appears to plot the same data as S3A, but P5 had zero values for HIV polyA across all T-cell subsets in S3A, but has the highest polyA measurement for Ttm in S3B. Are the data mislabeled? Also please explain the letters after the subsets (S3A x-axis) and the numbers after the participants in the legend of 3B. Are the latter the time points sampled? Finally, consider plotting S3B data on a log scale, and using nonparametric stats as some data are non-normally distributed.

The mistake has now been corrected. Average values are now plotted for those cell subsets that had more replicates.

7. line 218-219 "Most integrated proviruses contribute to the generation of swarms of replication-defective genomes in the affected individual [39-40]". This wording is confusing and misleading. I believe what the authors mean is that the vast majority of proviruses that persist during ART are genomically defective.

We thank the reviewer for the advice. We now rephrased the sentence accordingly (changes in *italics, text line 225*):

“Most integrated proviruses *persist for extended times as* replication-defective viral genomes in the affected individual.”

8. line 231 (and elsewhere). Authors refer to their bulk amplification and NGS sequencing of the V3 region as simply "NGS analysis" but this can be confusing as sometimes the provirus is the target, and other times it's viral RNA. In the case of line 231 ("QVOA positive wells"), it is unclear which is being assayed. It would be helpful if authors could clarify the target throughout.

We thank the reviewer for the helpful suggestion. For better clarity, we changed the phrasing in the respective text sections. Legends have been adjusted accordingly.

9. Figure S4. What were the criteria for determining the presence of replication competent HIV? It appears, for example that there are double-positive events in Ttm and Tem in participant 1. Also, please label the data for each participant on the figure itself, as they are not presented in numerical order.

The gatings for GERDA were chosen according to previous fine-tuning experiments (as described in Otte, Zhang et al., 2023) and each FACS run always included a negative, i.e. unstained control (see Fig S5F as a representative graph). As the co-detection of Env-signals remains quite challenging (e.g. due to the small number of Env molecules per virion or on the cell surface), double-positive events are only considered when Env and Gag levels are both sufficiently high. Thus, cells harboring replication-competent virus are considered those that have high Gag+Env+ events and in addition a confirmed positive viral outgrowth (X-Gal).

10. Figure S5. How were QVOA wells chosen for sequence analysis? Were these the wells plated at (estimated) limiting dilution - in which case the observed diversity can be interpreted as diversity generated in vitro - or were these wells that likely contained more than one reservoir cell (or both)? The S5A highlighter plot does not strongly support the "close relationship" between Tcm and B_3 sequences suggested by the tree - can the authors include bootstrap values to give the reader an indication of confidence in the topology? Also it appears that there may be alignment issues for some sequences (e.g. B_1_C1 or B_1_A14)?

For clarification: Wells were selected according to the results obtained in Figure 2 B, C. In the case of 2B (P1, TN cells) only light blue (5/7 positive wells) or orange (2/7 positive wells) variants were predominant. Accordingly, only one representative of the blue outgrowth wells (well No 1) and one representative for the orange variant (well No3) from TN fractions were chosen. The same procedure was applied for TCM.

As outlined in the M&M section, a fixed number of cells was used for setting up a VOA, as has been described also by others (Lorenzi J et al PNAS 2016 ). To better reflect the procedure, the term “QVOA” has now been changed to “VOA” in all text sections.

Variants B_1_C1, B_1_A7 or B_1_A14 were distant from all other variants due to gaps that were found near the 5’ end. These deletions have most resulted from erroneous amplification during the PCR process. As we considered this a technical limitation, the missing information was copied from the 5’ region of the corresponding SGA sequence B_1_C11 in the affected regions. This clarification has now also been added to the Figure legends and the M&M section.

The respective text has been adjusted. Changes in italics, text line 247:

“In Fig S5A, the first 1000 bp of the red and blue clades are almost identical, suggestive of a potential viral recombination event. All sequences share the same V3 region implying a common ancestral virus that has further evolved inside the host through mechanisms like recombination events, RT- or APOBEC-induced mutations.”

Reviewer #3: (No Response)

**Part III – Minor Issues: Editorial and Data Presentation Modifications**

Please use this section for editorial suggestions as well as relatively minor modifications of existing data that would enhance clarity.

Reviewer #1: Please see full review.

Reviewer #2: Minor comments

1. The first sentence of the last paragraph of the intro (lines 90-91) describes the study design and should go earlier e.g. before the summary of findings.

We agree with the reviewer and have now moved this paragraph of text to line 87.

2. Figure S1 legend says the numbers under the pies are numbers of "detected variants" but this cannot be right. Are these total NGS read counts?

The reviewer is, of course, correct and we apologize for the mistake. For better comprehension of the NGS data we have re-done Figures 1B, 2 and S1, which now indicate, how many sequences with a relative abundance of >1% and >0.1% were identified. We also indicate the total number of identified variants. We have added a value for “percent representation” of the most abundant variants to the graph. The unnecessary information about “total reading counts” has now been deleted to avoid an overload with numbers.

3. To respect current language guidelines, authors should use the term 'participant' or 'individual' throughout, and avoid the use of 'patient'

Thank you. We have replaced the term throughout the text.

Reviewer #3: (No Response)

PLOS authors have the option to publish the peer review history of their article ([what does this mean?](https://journals.plos.org/plospathogens/s/editorial-and-peer-review-process#loc-peer-review-history)). If published, this will include your full peer review and any attached files.

If you choose “no”, your identity will remain anonymous but your review may still be made public.

**Do you want your identity to be public for this peer review?** For information about this choice, including consent withdrawal, please see our [Privacy Policy](https://www.plos.org/privacy-policy).

Reviewer #1: No

Reviewer #2: No

Reviewer #3: No

**Reviewer 1 complete Review document**

HIV-1 diversity in viral reservoirs obtained from circulating T-cell subsets during early ART and beyond

Zhang and Otte...Klimkait, and the Swiss HIV Cohort Study

**Summary of manuscript:**

The manuscript here by Zhang and Otte *et al*. examined Env V3 diversity in various T cell subsets during early ART to characterize the dynamics over time. Very interestingly, the authors found a strong association between the Torque-teno-virus (TTV) and levels of HIV-1 plasma viremia and viral transcription as measured by polyA. Additionally, the level of Env V3 diversity appeared to reflect TTV load and was dynamic pre-ART and following therapy. The authors bring attention to a possibility of utilizing TTV load for immune status as it relates to HIV infection.

While intriguing, there were several major concerns that should be addressed in order to strengthen the manuscript as there is a great amount of potential (detailed below). Further, please be mindful of Person First language by replacing instances of “patient”.

**Specific comments (major and minor)**

Abstract:
Generally, the abstract reads well. The sentence in Line 25, “Complementing information was obtained...” seems worded oddly, possibly consider re-wording. While the abstract does not serve to explain all findings, the connection to TTV is lacking and/or weak. I found it slightly distracting as I had never heard of this virus before and thus, any clinical relevance was lost.

**Introduction:**

- Line 55: “HIV-1 virus reservoir”, the word ‘virus’ is repetitive to ‘HIV’.

The corresponding line has now been corrected.

- Line 57: Suggest changing to just “ART” as it is all highly active and capable of preventing ongoing rounds of viral replication.

Throughout the text, the abbreviation “ART” has now been used instead of “Antiretroviral therapy”

- Lines 59-63: Suggest rewording and making more succinct. It is understood what the authors are trying to say but the execution is disjointed. In addition, while of course rare, proviral eradiation has been shown to be possible through the Berlin Patient and other hom. CCR5delta32 alloHSCT.

We thank for the advice and have re-written the section; a statement about CCR5∆32 has been included. (Text line 62)

- Line 65: “Infection with HIV-1” suggest changing to “HIV-1 infection”.

Text has been changed accordingly (Text line 63).

- Line 65-68: Words like ’significant’ and ‘short time’ feel non-descriptors. Consider Codin (1995) PMID 7824947 as it also describes how the population size is a key factor.

We have adjusted the text accordingly. Changes in italics (Text line 63):

“*HIV-1 infection* is often characterized by the rapid development of a *genetically diverse viral landscape within months after primary infection*, which is attributable to the high error rate of the reverse transcriptase enzyme[16] and a rapid viral turnover[17]”

- Line 78: Avoid “patient” and use People First language.

We have replaced the term throughout the text.

- Lines 80-83: While still the introduction, include how you are measuring diversity.

We have now added a definition in lines 92:

“Individuals with highly diverse reservoirs were defined as those who exhibited a continuously changing viral reservoir >3 weeks after starting ART, characterized by more than 3 variants, each with a relative abundance greater than 5%, identified by NGS”

- Line 88-89: The TTV load correlate to HIV transcription as a suitable marker for clinical manifestation is too strong (discussed further in later comments), suggest modifying.

Please see Rev 2 major comment 2. After averaging all values for each individual, we had to re-analyze our data again (Fig.3). For poly-A measurements were not detectable for all individuals which made it hard to perform any statistical tests. Thus, we have removed the strong statements and just report certain “trends” in the discussion.

**Results:**

- Lines 99-100: I would specify “different phases of HIV infection”. Acute vs chronic, Fiebig staging, etc would be useful. Take the time to mention STable 1 since it would help the reader greatly.

We updated sTable1 where applicable and made a reference to the clinical information.

- Line 111: “chronic control situation” is worded oddly.

We rephrased the sentence, changes in *italics* (Text line 115):

“As a chronic *virally suppressed* control with a documented earlier acute infection phase, we included P9 and monitored from day 531 after treatment start (S1 Table).”

- Line 139-143: In relation to STable 2, please double check spelling, proper italicization when appropriate, and for drug names for ART and other treatments use the international naming (e.g. valganciclovir instead of valcyte).

STable 2 has been corrected accordingly as suggested.

- Line 150-154: Consider adding that viremia was suppressed to undetectable levels in donors following ART initiation, or something to that edect, it is worded oddly. It is mentioned that viral transcription was detected even post-ART for “long periods”, is there a reason that the authors might have thought otherwise since stochastic reactivation of infected cells contributes to residual viremia. The last sentence about transcriptional activity would be better connected without the statement of suppression of viremia between them.

Accordingly, we have now rephased corresponding paragraph (changes in *italics,* text line 155):

“*Intracellular v*iral transcription remained detectable for long periods (at least 745, 582, 811 or 443 days after treatment for the individuals, respectively). This *is suggestive of continuous* viral transcriptional activity in the viral reservoirs *despite* the absence of any detectable particle production or any release into the circulation, *where viral loads remained undetectable.”*

- Lines 159-: It is a little misleading to state you are interested in understanding viral dynamics in the long-term reservoir on ART. Perhaps something was missed here. But once on ART, ongoing rounds of viral replication is halted, so there would be a lack of evolution, and studies by Sarah Joseph and Zabrina Brumme have independently shown that the reservoir post-ART is primary reflective of the composition right before ART initiation—which the author’s data does support in Line 177-178. The idea of “long-term” would be >4 years, perhaps even longer (>7 years), or even longer. Donor 9 is the only that is even close to 4 years. It is also known that diversity is less in those who started ART during acute infection, in part due to the presence of a smaller reservoir size. It is not clear if these factors were considered. It was not clear in SFig 1 what “total” referred to, assuming total sequences obtained?

We agree with the Reviewer regarding the use of the term “long-term”. We have now rephrased it to be more precise (changes in *italics,* text line 164*)*:

“To understand the viral dynamics during *the early course of* therapy, we focused on changes in indicative Env V3 loop sequences of the proviral DNA and analyzed them by NGS over time under treatment.”

For better illustration of the NGS data, the pie plots shown in Fig 1B, 2 and SFig1 have now been updated (see also response Rev 2, minor comments #2).

- Line182-188: What are the subtypes of these donors? Assuming subtype B. But including that would be helpful to place the tropism prediction into context since Geno2Pheno doesn’t work well for all subtypes. Have the authors considered also using PhenoSeq (https://tools.burnet.edu.au/phenoseq/) to support their predictions through the use of two software tools? The authors mention that in P4 there was a “decrease” in X4 tropic viral variants, have these numbers been normalized to how many sequences were obtained (i.e. time point 1 has 50 sequences but time point has 500, the sampling depth at TP2 is 10x greater).

All individuals harbored a subtype B virus (which was added as info on S2 Fig), for which Geno2Pheno has been demonstrated to be a very reliable tool. The subtype information has now been added to the legend of S1 Fig. All tropism-related data are summarized as the sum of all relative abundances of X4 or R5-tropic viruses found at each time point. The updated Figure layout (including the total sequences obtained) makes it now easier to get a better understanding of sequencing depths. For the special case of P4 we have an ever-changing Coreceptor profile with a stable total variant identification count. Only for the last time point, the sampling is 10X less than for the second time point, but still not underrepresented compared to the other time points and respective identified sequences.

- Line195-203: Suggestion to clarify Figure 2 legend. In reading the Results text it was clear that the V3 sequences were from the proviral population but the legend begins talking about post-stimulation which is confusing. Also, are the Figures labeled correctly since there is “Fig 1B”? Also suggest adding the number of sequences obtained as a total for each subset and normalized if possible. If the bulk CD4 was used as a comparator, it would be nice to see some sort of p-value to understand how large some of these clones are between subsets compared to the bulk CD4.

We have now rephrased the paragraph (changes in *italics, text line 203)*:

“*Proviral* NGS *analysis* showed that in most individuals the *repertoire of identified variants in* the TN and TCM compartments *was largely similar to that identified for the bulk CD4+ T cells or for earlier time points* (Figs 1B and 2A). *For P1, we detected in TTMs a predominant proviral variant (90.4% of the whole viral population) that was only found as a small proviral minority in bulk CD4, TN, or TCM (Fig 2A). This small minority was already present for bulk proviral NGS sequencing during earlier treatment periods, e.g. on days 86 and 201 after therapy (Fig 1B*). The same phenomenon of *proportionately higher predominating variants* was seen in the TTMs of P2, P3, P4, and P7, and in the TEMs obtained from P1, P2, P4, and P5 (Fig 2A).
 The cells from individual P8 consistently possessed a very low proviral load, and even after 5 days post-stimulation an amplification of the env V3 region remained unsuccessful (S3 Fig).”

As pointed out for the text in lines 159 ff and Rev 2, minor comments #2, total variants, percentages >1%, >0.1% have now been included in the pie charts.

- Line 222-226: In following the author’s Methods section related to the measurement of polyA HIV transcripts there are two points that would be important to bring up. The reference (#30) for the method of HIV polyA RNA is the reference for TTV detection. I have assumed the authors are using an HIV-1 gene-specific polyA primer for the purposes of the results. While the amount of RNA is normalized to CCR5, it would be important to normalize to the number of infected cells. Without knowing how many infected cells were assayed, in the cases where it wasn’t detected, enough infected cells might not have been assayed. Also it might be worth noting that depending on the length the RNA sat in the -80C, you can have degradation starting after several weeks/months.

We thank the reviewer for identifying this misplaced reference. The reference for HIV poly-A RNA quantification has now been correctly used here.

For a more comprehensive and concise estimation of the transcription competent HIV-1 reservoir we have now added information about HIV polyA/ HIV DNA in STable 1. Fig 3 B has been updated accordingly.

Intracellular RNA was in all cases extracted from DMSO-containing N_2_-cryo-preserved cell samples that are routinely stored for the SHCS. All extracted RNA species were either directly quantified or were stored immediately at -80°C for subsequent quantification soon after extraction (≤ 1-2 weeks).

•	Line 230: For the IUPM 95% C is only need to report to the 2nd decimal place (also Line 243). Out of curiosity, and might be worth including, was the viral outgrowth performed at limiting dilution, or multiple bulk wells plated? Could be worth including as well to be clear whether the outgrowth assay was a quantitative VOA—especially since you state QVOA in Line 249.

All confidence intervals have now been corrected accordingly, and the term “QVOA” has been replaced by “VOA” throughout the entire text for higher clarity.

- In Line 245, I think the authors mean S2B not S2F.

Thanks for pointing out the wrong Figure numbering. We have corrected it to “S1F”, corresponding to the matching RNA NGS data during unsuppressed time periods of P07.

- After reading through the legends and methods, a suggestion that would benefit your phylogenetic trees would be the following. The gaps are artificially leading to long branches. If possible, either gap-strip manually, then reconstruct the trees. Or if using MEGA, try performing a ‘complete deletion’ NJ. Also change the scale to nucleotide so the reader can easily determine how many bases different the sequences (or populations) are from one another. Also looking at S5B, the viruses that have the common “A” are probably clonal. The random 1-2 nucleotides observed to give them little branches is probably fuzz from introduced sequencing error.

We thank the reviewer very much for this helpful and useful suggestion. As also pointed out in the response to Rev 2 comment #10, we have now corrected the long branches of B_1_C1, B_1_A7 or B_1_A14. As these most likely stem from erroneous PCR amplifications, the 5’ region of the similar SGA sequence B_1_C11 has now been inserted at the affected regions. This modification has also been added to the Figure legends and the M&M section.

- Line 256: “derailment” is an odd word to use here.

Sentence has been rephrased (changes in *italics, text line 265)*:

“To investigate aspects of the HIV-*induced dampening* of crucial immune functions during early periods after diagnosis, we compared CD4+ and CD8+ T cell populations in individuals with high variability to those with only a few viral variants in the circulating blood.”

- Line 258: The authors need to define what is being set as “high variability” as it is quite ambiguous. If the cut-od is >5 viral variants, please state that, or if the authors can develop a statistical determination, use that as well. But as a reader, knowing what exactly is meant is crucial.

Changes have been included in the manuscript (as mentioned earlier (Line 92), see comment for lines 80-83
“Individuals with highly diverse reservoirs were defined as those who exhibited a continuously changing viral reservoir >3 weeks after starting ART, characterized by more than 3 variants, each with a relative abundance greater than 5%, identified by NGS.”

- Line 270: For reported p-values in-text and in the figures, too many sig figs are used, the authors can simplify them.

The sentence has now been simplified after re-analysis of averaged data (see Rev#2 major comment2), with changes in *italics, text line 281*:

“With the onset *of ART*, TTV loads decreased in the group of individuals presenting with a high genetic HIV diversity *in the first year and stayed stable in the following years*, while in the low diversity group, TTV loads remained stable independently of the individual treatment situation.”

- Line 263-280: The inverse correlate of TTV and immune status is fascinating. As stated previously, the leap to an indicator and having clinical significance is a little strong and does require more work. The biggest concern I have here is that if you sampled TTV in a person living without HIV overtime, would the dynamics of TTV load be similar as those in persons living with HIV—since it is highly prevalent? And if that work has already been done, the authors should highlight that before going into potential differences of high and low diversity groups. It would also benefit to demonstrate data where TTV is not detected. It begins to fall outside the scope of this article, but it feels like a strong emphasis is drawn to it. By including additional experiments such as suggested, understanding the possible correlation and overall biological significance can better be explored.

We intend to emphasize here that TTV is currently being used in some diagnostic settings for determining the general immune status in transplantation settings (De Vlaminck I et al 2013 Cell; Fernández-Ruiz M et al 2019 Am J Transplant; Gore EJ et al 2023 FMed) e.g. with “TTV R-GENE” by Biomérieux. The prevalence of TTV in the human population is very high and thus renders it a good independent marker of immune correlates. As suggested by Rev 2 major comment #2, we had to re-analyze the data using averaged values for each patient. After our thorough re-analysis, we found a trend for different CD4 levels between the high and low diversity group. For polyA and for TTV quantification too few data points were available to allow a conclusive statement.

We have now softened the statement in the text as our cohort is rather small not allowing to draw general, absolute conclusions.

**Discussion:**

- Line 309-315: As stated, prior, the strong language of TTV is too much. The authors connect alloHSCT with TTV but the reference to Einkauf *et al.* does not seem appropriate here.

We changed the respective paragraphs on TTV and also toned it down after our more recent re-analysis. The reference was updated as well.

- Line 348-354: This comment originates from the introduction, and this appears to be a “good location” to discuss. The authors are correct in their assessment on central memory. Have the authors normalized the different subsets since central memory is much larger than effector memory? In the introduction, it was expressed as though this was a “new” finding but Chomont...Sékaly (2009) and Kwon...Siliciano (2019) also highlighted these points regarding central memory. This would go further to help Lines 254-259 as well.

We agree that the finding on TCM is not entirely new, which is why we have also cited the Article of Chomont et al 2009. However, our argument addresses the anatomical localization of the memory T cell pool. In this regard, we do not only find TCM relevant but also TN, which are both mainly found in draining Lymph nodes according to their high expression levels of CCR7/CD62L. TTM/TEM, however, are patrolling at more distal sites.

We have now included data on the absolute cell numbers and their respective contribution to the memory compartment. This graph has been included as supplemental FigS6. Although naïve T cells are higher in numbers, TCM, TTM and TEM are in comparable ranges to each other. See also Rev 2 major comment 2 for details. A paragraph on the limitations of the study has been added to the discussion section, text line 379:

“Besides using a larger number of samples, the analysis would principally benefit from using higher blood volumes to increase the chances of reactivating dormant proviruses. Yet, the Swiss cohort ethical approval only allows full blood volumes of up to 40 mL. Irrespectively, TCM (mean 12.30%), TTM (mean 8.49%) and TEM (mean 20.53%) are similarly represented with equal normalized proviral and transcriptional loads (Figs S3B and S6).”

- Line 385-387: This sentence does not make, the concept of what the authors are trying to say is there but could use some work.

Rephrased sentence, changes in *italics, text line 401*:

“*Follow-up studies should include bigger cohorts to study further* the combination of HIV-1 transcription levels and plasma TTV loads *to* provide *potentially* useful indicators for obscure HIV activity in viral reservoirs.”

**Methods:**

- If the authors can include the purity% of their subsets as a supplemental table or elsewhere, it would be a good addition. Or a general statement of the >x% purity etc.

The strict limitation in the amount of cell material posed an absolute limitation. Because of this restriction and to be reproducible throughout the study, all workups strictly applied established magnetic bead-based methodologies for the harvest and purification of the cell types of interest. Although we are fully aware that this does not lead to a 100% pure cell fractions, the high enrichment is largely accepted in the field. This is further confirmed by the findings shown here that the applied cell-type separation leads to the identification of well-separated and quite different virus populations.

- Could more be added about the pipeline for NGS? And the Illumina runs? Assuming dogtags are used, is it possible to bin sequences and generate majority-rule consensuses similar to what has been reported previously the Swanstrom group? Overall, since Geneious’s assembly methods can vary, some additional detail would be helpful.

We thank you for the detailed question about NGS. All NGS were conducted by an experienced diagnostic NGS service partner. All processing of the data was done using a validated pipeline called “deepType-HIV”. The respective information has now been added to the M&M section.

We are not familiar with “dogtags” but assume that the reviewer asks for the use of specific primer-IDs: We have not used those, as this is not possible in combination with the Nextera sequencing approach (random barcodes are only at the amplicon ends, by fragmenting the amplicons we lose the information for most reads).

- For the statistics, is there a particular reason the authors did not use Spearman— seemed more logical. Also, it would be good to state what type of “regression” was performed. Was the data normal so that a linear regression (assuming that was the method) made sense without data transformation? For simplicity, you could easily remove the 95%CIs of the regression.

Please see Rev 2 major comment 2 and comment in Line 88-89.
